# Role of HSV-1 Capsid Vertex-Specific Component (CVSC) and Viral Terminal DNA in Capsid Docking at the Nuclear Pore

**DOI:** 10.3390/v13122515

**Published:** 2021-12-15

**Authors:** José Ramon Villanueva-Valencia, Efthymios Tsimtsirakis, Alex Evilevitch

**Affiliations:** Department of Experimental Medical Science, Lund University, 228 14 Lund, Sweden; jose.villanueva@med.lu.se (J.R.V.-V.); efthymios.tsimtsirakis@med.lu.se (E.T.)

**Keywords:** capsid vertex-specific component (CVSC), HSV-1, nuclear pore complex, UL25, UL36, DNA ejection

## Abstract

Penetration of the viral genome into a host cell nucleus is critical for initiation of viral replication for most DNA viruses and a few RNA viruses. For herpesviruses, viral DNA ejection into a nucleus occurs when the capsid docks at the nuclear pore complex (NPC) basket with the correct orientation of the unique capsid portal vertex. It has been shown that capsid vertex-specific component (CVSC) proteins, which are located at the twelve vertices of the human herpes simplex virus type 1 (HSV-1) capsid, interact with nucleoporins (Nups) of NPCs. However, it remained unclear whether CVSC proteins determine capsid-to-NPC binding. Furthermore, it has been speculated that terminal DNA adjacent to the portal complex of DNA-filled C-capsids forms a structural motif with the portal cap (which retains DNA in the capsid), which mediates capsid-NPC binding. We demonstrate that terminal viral DNA adjacent to the portal proteins does not present a structural element required for capsid-NPC binding. Our data also show that level of CVSC proteins on the HSV-1 capsid affects level of NPC binding. To elucidate the capsid-binding process, we use an isolated, reconstituted cell nucleus system that recapitulates capsid-nucleus binding in vivo without interference from trafficking kinetics of capsids moving toward the nucleus. This allows binding of non-infectious capsid maturation intermediates with varying levels of vertex-specific components. This experimental system provides a platform for investigating virus–host interaction at the nuclear membrane.

## 1. Introduction

For most DNA viruses and a few RNA viruses, the central virus–host interface during the course of infection is the nuclear membrane, which controls the penetration of viral genome into the host nucleus, subsequently leading to infection [1,2,3]. Many viruses bind at the nuclear pore complexes (NPCs), which facilitate viral genome uncoating and its transport into the nucleus, where genome replication and viral assembly take place [1,2,3]. However, despite virus–NPC binding being a significant part of the viral infectious cycle, the molecular details of this interaction are poorly understood for most viruses, including herpesviruses. In this work, we explore the role of capsid surface structural motifs in docking to NPCs, the key step required for ejection of viral genome into a host nucleus. We use human herpes simplex type 1 virus (HSV-1) as a prototype of the nine known human herpesviruses, which affect billions of people worldwide [4]. Specifically, we answer a long-standing question of whether the terminal part of the viral encapsidated DNA that is adjacent to the portal is required as a structural element for NPC docking [5,6,7,8]. We also investigate the role of capsid vertex-specific component (CVSC) proteins, which are located at the twelve vertices of the HSV-1 capsid and have been found to interact with nucleoporins (Nups) of NPCs [9,10,11], in mediating capsid docking to the NPCs.

Herpesviruses consist of a double-stranded (ds) DNA genome packaged within an icosahedral capsid that is surrounded by an unstructured protein layer, the tegument, and a lipid envelope. After binding at the outer membrane, viruses enter the cell cytoplasm and are transported toward the nucleus along the microtubular network. The viral capsid ejects its genome upon docking to an NPC, which forms a passageway for molecular traffic into the nucleus [5,6,7,8,9,10,11,12]. We recently showed for HSV-1 that the tens of atmospheres of genome pressure are responsible for DNA ejection into a host cell nucleus, causing infection [13]. After herpes capsids eject DNA into the nucleus, viral genomes rapidly replicate during a lytic infection [8]. It has been observed that docking of HSV-1 at the NPC is mediated by the minor capsid protein UL25, which binds Nup214 situated on the cytoplasmic NPC surface [10,11], and by tether tegument protein UL36, which binds Nup358 localized on the cytoplasmic NPC filaments [9,14] (gene names are used to refer to their respective proteins). It was suggested that these interactions ensure alignment of the capsid portal with the NPC opening pore, facilitating DNA ejection into the nucleoplasm [15]. The HSV-1 capsid is comprised mainly of the major capsid protein VP5 (UL19), organized in 11 pentameric (penton forming vertices) and 150 hexameric (hexon) capsomer subunits [16]. The twelfth vertex is occupied by a portal complex that is formed from a dodecameric ring of UL6 protein; the portal complex serves as the portal channel via which DNA is packaged and it is essential for cleavage of replicated viral DNA into the preformed capsid [17]. The capsomers are stabilized by the triplex heterotrimers, composed of two VP23 (UL18) proteins and one VP19C (UL38) protein, which assemble at all three-fold and quasi three-fold axes [18]. HSV-1 capsid assembly begins in the infected cell nucleus with spherical precursor particles called procapsids assembling around a protein scaffold [19,20,21]. The scaffold protein is proteolytically cleaved and removed during DNA packaging, and DNA-containing C-capsids are formed [18,22,23]. The insertion of DNA and expulsion of scaffold proteins during capsid maturation is accompanied by angularization of the capsid and outward extension of the DNA packaging portal, which is driven by DNA pressure buildup [24]. Capsid maturation is also associated with addition of minor capsid proteins [22]. C-capsids mature into infectious virus particles after being transported out of the nucleus and acquiring tegument proteins and lipid envelope during transport [25,26]. C-capsids are essentially identical to the virion capsid structure with the exception of the capsid-associating tegument proteins [27]. Along with C-capsids, two dead-end products, A- and B-capsids, are also formed during virus particle assembly. B-capsids retain cleaved scaffolding proteins, do not initiate DNA packaging and do not complete the maturation pathway [25,27]. A-capsids lack scaffold and will initiate DNA packaging but fail to retain DNA [25].

A-, B-, and C-capsids are structurally identical with regard to the number of major capsid proteins, portal forming proteins (UL6), and triplexes (with the exception of the scaffold protein retained in the B-capsids). The major difference, however, lies in the abundance of the proteins comprising the CVSC. CVSC consists of minor proteins: a singlet of UL17 and duplexes of both UL25 and UL36, which is a tegument protein that might be involved in recruitment of other tegument components to the mature capsid [17]. CVSC proteins are present at all 12 capsid vertices, including the portal (although CVSC protein number is higher at the portal [5,17]). UL17 and UL25 subunits form a star-shaped density that extends from the top of each VP5 penton to the adjacent VP23/VP19C triplexes and VP5 hexons, stabilizing the overall capsid structure [28]. CVSC proteins also interact with UL6 portal proteins, forming a cap preventing spontaneous release of DNA from the capsid [17,29]. UL36 and UL25 are cleaved upon capsid docking to the NPC, which was suggested to facilitate DNA ejection [11,14,17]. While UL36 and UL25 have been shown to interact with Nups within the NPC, it has not been demonstrated whether they are essential for initial capsid-NPC docking [9,10,11,14]. It has been challenging to investigate whether CVSC proteins are essential for NPC-capsid binding because the deletion of either of these proteins interferes with viral assembly [11,30].

Fortunately, the HSV-1 capsid assembly process offers an ability to isolate B- and A-capsids, dead-end products of C-capsid assembly. While B- and A-capsids are not considered assembly intermediates (they are stably co-purified with C-capsids), they directly reflect the intermediate states of capsid maturation toward C-capsid with associated progression of CVSC protein occupancy at capsid vertices, which in turn facilitates the mechanical stabilization required for capsids to package pressurized DNA [28,31,32,33,34]. Specifically, the level of CVSC proteins capsid occupancy has been found to follow C > A > B progression [17,18,19,35,36,37,38,39,40]. Thus, targeting the NPCs with A-, B-, and C-capsids offers an opportunity to elucidate how varying levels of UL25/UL36 on the capsid surface impact capsid-NPC binding. Since A- and B-capsids are lacking viral DNA, we also show the role terminal DNA plays in capsid-NPC docking. This experiment, however, is not feasible in cells since A- and B-capsids do not leave the nucleus during viral replication [25,27]. In this work, we circumvent this problem by using cell-free reconstituted cell nuclei incubated with purified A-, B-, and C-capsids, respectively, to investigate capsid binding ability at the NPCs on a nuclear membrane.

## 2. Results and Discussion

Purified GFP-labelled A-, B-, and C-capsids (green, strain K26GFP, HSV-1 strain expressing GFP-tagged VP26 protein, see Section 3), respectively, were added at the same concentration to reconstituted cell nuclei and incubated at 37 °C in capsid binding buffer (CBB). To ensure that A-, B-, and C-capsids were added to nuclei at the same concentration, the fluorescent GFP-signal from each capsid type was measured with a fluorometer and capsid concentrations were adjusted to the same fluorescence signal intensity (see Section 3). Nuclei were isolated from rat liver cells (see Section 3) and reconstituted in a cell cytosol solution with added ATP-regeneration system. Both of these cellular components are required for effective capsid binding to NPCs and opening of the NPC channel for viral DNA translocation (cytosol contains importin-β, which is required for efficient HSV-1 capsid binding to NPCs) [13,41,42]. The number of virus capsids added per nucleus was optimized to correspond to the biologically relevant multiplicity of infection, MOI ~100 pfu/cell during reactivation of latent HSV-1 infection in trigeminal ganglia (TG) neurons [43,44,45] (MOI refers to the infectious viral particles approximately corresponding to the viral capsids able to bind nuclei and eject their genomes [46]).

**Figure 1:**
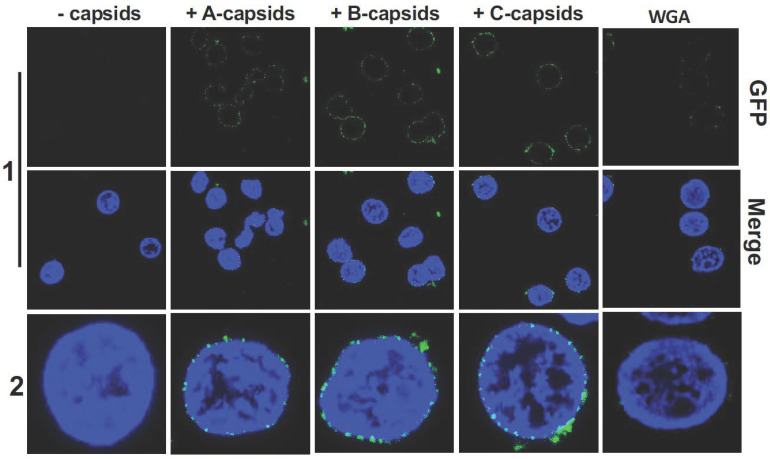
Imaging of reconstituted capsid-nuclei system confirms specific binding of A-, B-, and C-capsids to the NPCs at the nuclear membrane with similar occupancy. Confocal fluorescence microscopy images show binding of GFP-HSV-1 A-, B-, and C-capsids (green) to DAPI-stained isolated nuclei (blue), in the presence of cytosol and ATP-regeneration system. The addition of wheat germ agglutinin (WGA) reduces capsid binding to nuclei for all three capsid types, which demonstrates that capsids bind specifically to NPCs as opposed to binding anywhere on the nuclear membrane. One representative image of C-capsids and WGA-treated nuclei is shown.

**Figure 2:**
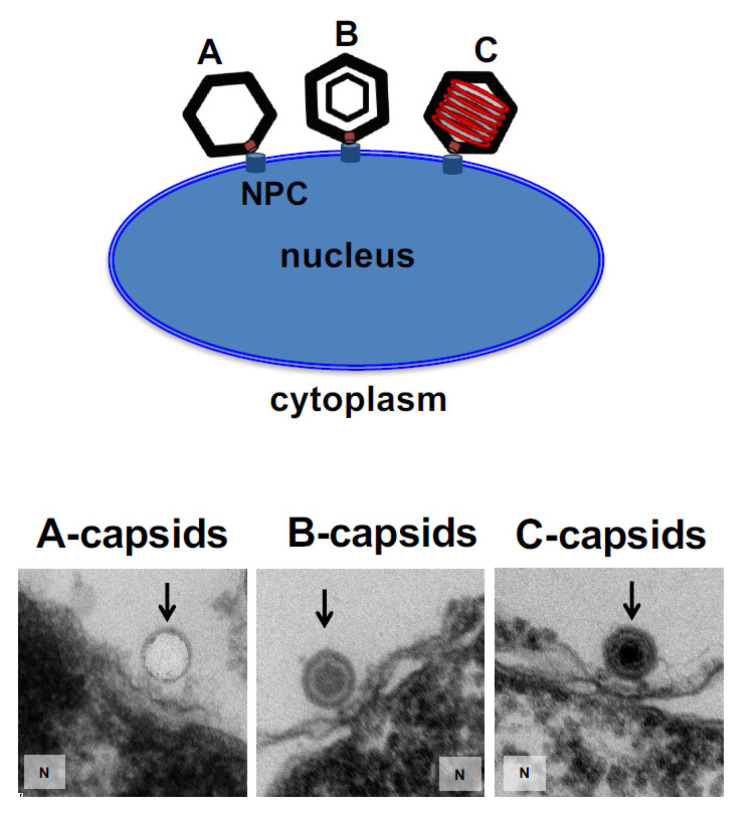
Ultra-thin sectioning EM shows binding of A-, B-, and C-capsids to the NPC basket structure at the nuclear membrane of reconstituted cell-free nucleus. The observed density inside the C-capsids corresponds to packaged DNA before it is ejected into the nucleus since sample was kept at 4 °C prior to fixation while DNA ejection occurs at 37 °C [13].

Figure 1 shows confocal fluorescence microscopy of GFP-labelled HSV-1 A-, B-, and C-capsids bound at the surface of DAPI-stained cell nuclei (blue). To demonstrate that capsids bind specifically to NPCs (as opposed to anywhere on the nuclear membrane surface), we used wheat germ agglutinin (WGA). At high doses (0.1–0.5 mg/mL), WGA associates with the glycoproteins within the NPC with high affinity [41,47,48] and reduces capsid binding, demonstrating capsid-NPC binding specificity, see Figure 1 (WGA reduces binding of A-, B-, and C-capsids, respectively, and one representative image of C-capsids and WGA treated nuclei is shown). We found that all three capsid types bound to NPCs on the nucleus surface, as observed in representative fluorescence images in Figure 1. It has been speculated that terminal DNA adjacent to the portal complex of DNA-filled C-capsids forms a structural motif with the portal cap (which retains DNA in the capsid), which mediates capsid-NPC binding. Our demonstration of A- and B-capsids (lacking DNA) binding to the NPCs shows that terminal DNA does not mediate capsid-NPC binding. As a complementary approach, we also visualized capsid binding to NPCs by ultrathin-sectioning electron microscopy (EM), which further confirmed docking of capsids to the NPCs. Figure 2 shows EM micrographs of A-, B-, and C-capsids bound to the NPC basket on a nucleus surface (the observed density inside the B-capsids corresponds to scaffold protein and the observed density inside the C-capsids corresponds to packaged DNA before it is ejected into the nucleus since the sample was kept at 4 °C prior to fixation, while DNA ejection occurs at 37 °C [13]).

**Figure 3:**
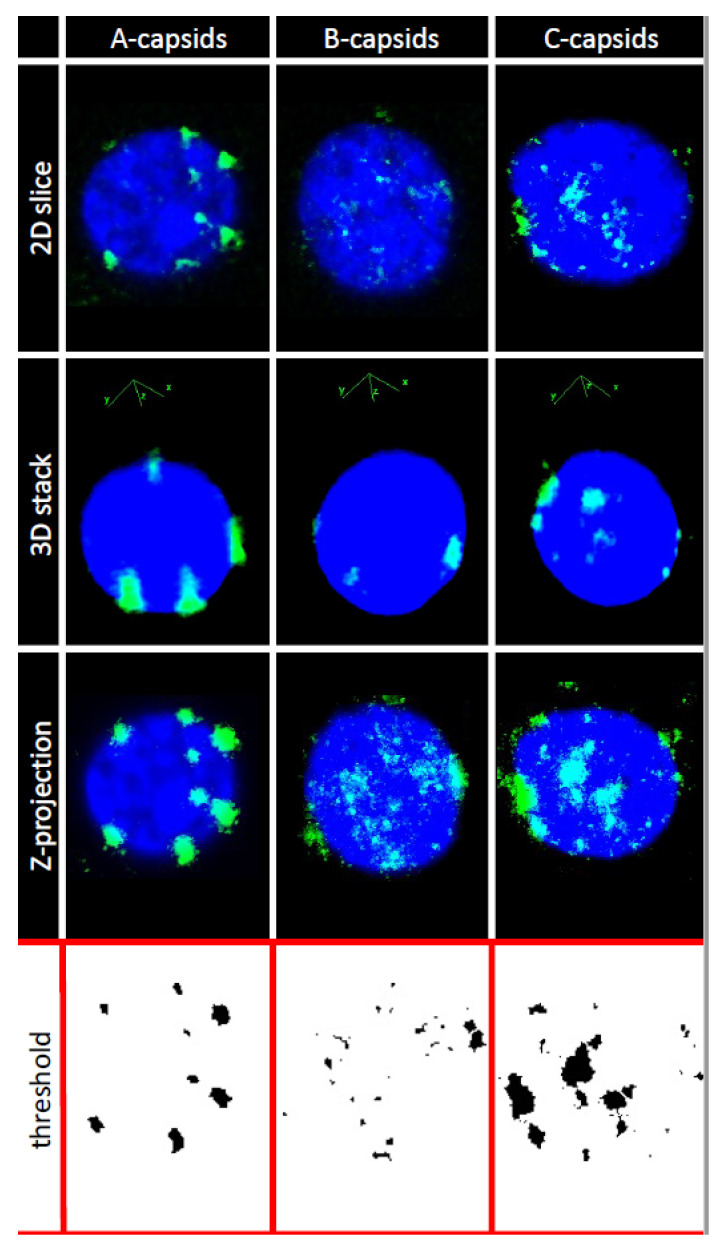
Representative images of reconstituted isolated nuclei with bound A-, B- or C-capsids. The rows show the general procedure (as described in Materials and Methods) to quantify, from a set of stacks, the number of capsids attached to nucleus surface. Steps of confocal fluorescence image analysis are shown for quantification of capsids per isolated cell nucleus. First row is a 2D slice (one focal plane) from the 3D stack GFP capsids (green) on DAPI stained nucleus (blue) in the second row. Third row is a Z-projection in 2D of all confocal plane slices in the 3D stack that was used for quantification of individual capsids, built using non-overlapping green pixels with the highest intensity from each slice (corresponding to capsids). Z-projection shows both channels and exhibits the projection of all capsids on a nucleus. The green channel was converted to a binary image using a threshold, as shown in the fourth row, in order to accurately compute number of capsids bound at each nucleus. Capsids outside of the nuclear membrane surface are not counted (as seen in the threshold images). Some nuclei showed capsid aggregates at the surface. Number of capsids in each aggregate was quantified using number of pixels corresponding to an individual capsid applied only to the aggregate surface area immediately at the nuclear membrane, limiting aggregate height to layer thickness corresponding to one capsid diameter. Automated capsid counting using these boundary conditions was performed with ImageJ software script. At least 50 nuclei were quantified for each sample.

**Figure 4:**
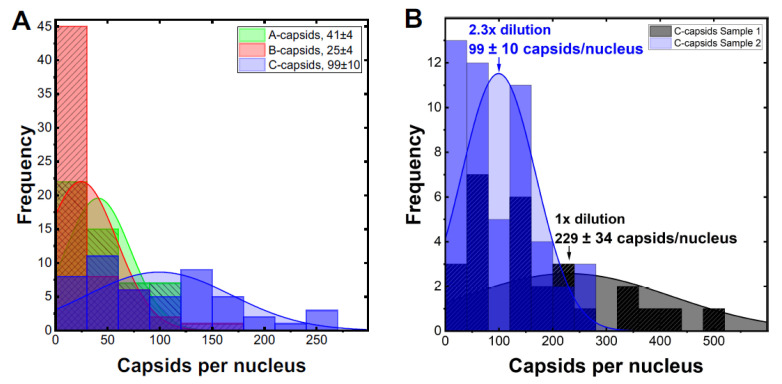
(**A**) Histograms showing the distribution of the computed number of A-, B-, and C-capsids, respectively, docked at the membrane of a reconstituted isolated nucleus. (**B**) Histogram demonstrating that ~2.3 times dilution of C-capsids translates into a ~2.3 times lower number of capsids bound per nucleus. This validates reproducibility of our capsid/nucleus counting approach. At least 50 nuclei were quantified for each type of capsids as well as for each dilution. The fitted Gaussian distributions provide the mean value of capsids/nucleus as well as the standard error.

**Figure 5:**
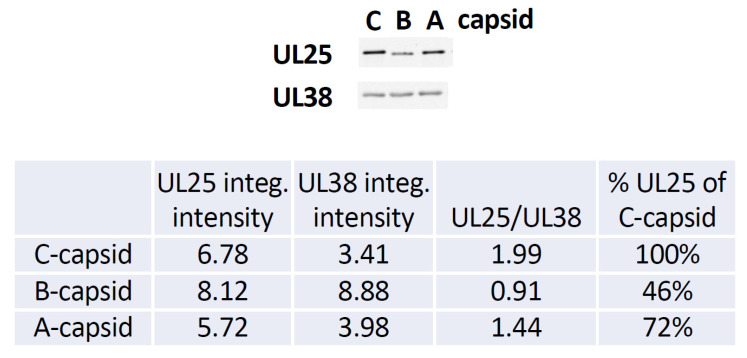
Quantitative western blot analysis of UL25 bound to wild-type (wt) KOS A-, B- and C-capsids. The membrane was probed with mouse anti-UL25, then stripped and reprobed with rabbit anti-UL38 as a measure of the load. The percentage of UL25 bound to each capsid type is shown relative to UL25 bound to wt C-capsid was (calculated through the normalized ratios of UL25 (63 kDa) intensity to UL38 (50 kDa) intensity on the blot. Data courtesy: Jamie B. Huffman, University of Pittsburgh.

In order to investigate whether CVSC proteins UL25 and UL36 play a central role for capsid docking to the NPCs, we quantified, using ImageJ analysis software, the average number of A-, B-, and C-capsids bound per nucleus circumference in confocal fluorescence images of GFP-tagged capsids docked to nuclei. We collected 3D stack images of nuclei with attached capsids using confocal fluorescence microscopy for GFP-HSV-1 capsids bound to DAPI stained nuclei, see Figure 3. We used Z-projection (2D) of the 3D stack to quantify individual capsids, averaging over at least 50 nuclei (and thousands of capsids) for each sample, see Section 3. Some nuclei had visible capsid aggregates which were converted to a number of individual capsids, using number of pixels corresponding to a single capsid at the nuclear membrane surface. Figure 4A shows the histogram distribution of A-, B- and C-capsids/nucleus. Gaussian distributions for collections of capsids bound per nucleus were fitted to obtain the standard error (SE). We obtained following levels of capsids bound per nucleus relative C-capsids (~99 ± 10 capsids per nucleus): C-capsids 100 ± 10%, A-capsids 41 ± 6%, B-capsids 25 ± 5%. Figure 4B shows a histogram demonstrating that ~2.3 times dilution of C-capsids, added to nuclei solution, translates into a ~2.3 times lower number of capsids bound per nucleus. This validates reproducibility of our capsid/nucleus counting approach and also confirms that selected capsid concentration does not oversaturate nucleus binding capacity. At least 50 nuclei were quantified for each type of capsids as well as for each dilution. It can be concluded from this data that. while all three types of capsids are binding to nuclei, the capsid binding efficiency is following the progression C > A > B. Significantly reduced levels of both UL25 and UL36 in B-capsids were previously reported [17,49,50]. It has been proposed that conformational changes in the capsid associated with DNA packaging expose UL25 binding sites, leading to a higher copy number of UL25 on the surface of C- and A-capsids compared to that of B-capsids (which do not package DNA) [38]. Somewhat different relative amounts of CVSC proteins on three HSV-1 capsid types have been reported in the literature, depending on the purification procedure used (since a portion of CVSC proteins is lost in the course of purification [51]). However, the relative amounts were always found to follow the progression C > A > B [17,19,35,36,37,38,39], in agreement with our data for the extent of capsids bound to nucleus. In Figure 5, we show a quantitative western blot (WB) analysis of relative amount of UL25 on A-, B-, and C-capsids using capsid purification protocol used in this study (see Section 3). UL38 capsid protein band was used as a reference to normalize each lane with the amount of loaded capsids, since UL38 capsid copy number is constant regardless of UL25 occupancy variation in a capsid. The WB data confirms the previously reported relative progression of capsid-bound UL25 amount relative C-capsids: C-capsids (100% UL25), A-capsids (72% UL25), and B-capsids (46% UL25). As mentioned above, determining the relative amounts of UL25 and UL36 on the capsids in a meaningful way is technically challenging (if possible). Some of UL25 and most of UL36 are lost during capsid purification process due to weak capsid attachment [17] (it was noted that UL36 is present at much lower levels on all capsids when compared to virions [17]). In Figure 5 we only show the relative amounts of UL25, since UL36 does not transfer well on the WB membrane due to its large size and there are no optimum antibodies for quantitate staining analysis (private communication with Jamie Huffman, University of Pittsburgh). However, UL36 binds to UL25 (as well as to UL17) [17] and its copy number on the capsid is likely to corelate with that of UL25. It can also be mentioned that UL36 mainly localizes into the cytoplasm and is added to capsids as they exit the nucleus [52] and translocate to a cytoplasmic compartment for final envelopment [30]. Hence, only small levels of bound UL36 were detected on A-capsids and almost no UL36 was detected on B-capsids [17,49,50].

Combined, our observations suggest that reduced levels of CVSC proteins on A- and B-capsids compared to that of C-capsids, result in lower capsid-NPC binding. Our findings are supported by recently published data for Kaposi’s sarcoma-associated herpesvirus (KSHV) showing that KSHV capsids (with capsid structure homologous to HSV-1 capsids) dock at the NPCs even when portal-ring-forming protein ORF43 is deleted (ORF43-null KSHV capsids) [15]. Based on this result, authors suggest that capsid-NPC binding is mediated by CVSC complexes at non-portal penton vertices (this type of non-portal binding results in infection “dead-end” particles since DNA cannot be ejected) [15]. In our study, however, we provide a direct evidence of CVSC mediated HSV-1 capsid docking at NPCs. It should be noted that UL6 portal ring structure or higher copy number of UL25 proteins capping the portal [17] could also mediate capsid-NPC binding, with higher affinity for the portal vertex than for the other 11 capsid vertices [11,17]. We found earlier that ~70% of C-capsids incubated with reconstituted cell-free nuclei ejected their DNA into nuclei [13]. If all twelve capsid vertices had equal NPC affinity, the fraction of capsids that ejected their DNA would have been 1/12 or ~8%. The fact that only 30% of capsids did not eject their DNA into a nucleus through the NPCs was explained by observation of several layers of capsids attached on top of capsids docked at the NPCs, preventing portal docking to the NPC, thus blocking DNA ejection. This also suggests that in addition to CVSC mediated capsid-NPC binding, HSV-1 capsid docking is not a stochastic event but is driven to the correct capsid orientation by the unique portal vertex structure, which has preferential binding affinity for the NPC. In the future study, we aim to investigate NPC-capsid binding of UL6 null and CVSC null mutants using the cell-free reconstituted nucleus system.

## 3. Materials and Methods

Cells and viruses. African green monkey kidney cells (Vero; ATCC CCL-81 from American Type Culture Collection, Rockville, MD, USA) and BHK-21 cells (ATCC CCL-10; from American Type Culture Collection, Rockville, MD, USA) were cultured at 37 °C in 5% CO_2_ in Dulbecco’s modified Eagle’s medium (DMEM; Life Technologiesk, Carlsbad, CA, USA) supplemented with 10% fetal bovine serum (FBS; Gibco, Waltham, MA, USA), 2 mM L-glutamine (Life Technologies), and antibiotics (100 U/mL penicillin and 100 μg/mL streptomycin; Life Technologies). The KOS strain of HSV-1 was used as the wild-type strain. The K26GFP HSV-1 recombinant virus (gift from Dr. Prashant Desai), which carries a GFP tag on the capsid protein VP16, was used in fluorescence studies. All viruses were amplified on Vero cells, and titers were determined on Vero cells by plaque assay. Viral plaque assays were carried out as follows: Viral stocks were serially diluted in DMEM. Aliquots were plated on 6-well trays of Vero cells for 1 h at 37 °C. The inoculum was then replaced with 40% (*v*/*v*) carboxymethylcellulose in DMEM media. HSV-1 plaque assays were incubated for 3–4 days. The monolayers were stained for 1 h with crystal violet stain (Sigma-Aldrich, St. Louis, MO, USA). After removal of the stain, the trays were rinsed with water and dried, and plaques were counted.

HSV-1 nuclear capsid isolation. Vero cells were grown to confluence and infected with K26GFP HSV-1 strain at a multiplicity of infection of 5 PFU/cell for 20 h at 37 °C. Cells were scraped into solution and centrifuged at 3500 revolutions per minute (rpm) for 10 min in a JLA-16.250 rotor. The cell pellets were resuspended in PBS (1.37 M NaCl, 27 mM KCl, 43 mM Na_2_HPO_4_·7H_2_O, 14 mM KH_2_PO_4_), pooled, and again centrifuged at 3500 rpm for 10 min. This washed cell pellet was resuspended in 20 mM Tris buffer (pH 7.5) with protease inhibitor cocktail (Complete; Roche, Basel, Switzerland) and incubated on ice for 20 min to swell the cells. The swollen cells were lysed by addition of 1.25% (*v*/*v*) Triton X–100 (Alfa Aesar, Haverhill, MA, USA) for 30 min on ice. Samples were centrifuged at 2000 rpm for 10 min and the resulting nuclei pellet was resuspended in a small volume of TNE (10 mM Tris, 0.5 M NaCl, 1 mM EDTA) buffer with protease inhibitor cocktail. Nuclei were disrupted by sonication for 30 s (in 10 s intervals, iced between rounds) and large debris were cleared by brief centrifugation (maximum speed for 30 s). MgCl_2_ and DNase I were added to the supernatant to 20 mM and 100 μg/mL, respectively, and the sample was incubated at room temperature for 20 min. The supernatant was then centrifuged at 11,750 × *g* for 90 s to pellet large debris, and further cleaned of small debris by underlaying with a 3 mL cushion of 35% sucrose-TNE and centrifuging at 23,000 rpm for 1 h. The capsid-rich pellet was resuspended in TNE + protease inhibitor cocktail, then loaded onto a 20–50% (*w*/*w*) TNE sucrose gradient and centrifuged at 24,000 rpm in a SW41 rotor for 1 h. The A-, B-, and C-capsid bands were extracted by side puncture, diluted at least 3X in TNE buffer and finally centrifuged at 24,000 rpm for 1 h to pellet the capsids. Capsids were gently resuspended in TNE and stored at 4 °C. The purification steps for mutant viruses were the same as described for KOS strain.

Rat liver nuclei isolation and cytosol preparation. Nuclei from rat liver cells were isolated as adapted from previously described protocol [41]. The intactness of nuclei was confirmed by light microscopy, EM (electron microscopy) and FM (fluorescence microscopy) by staining the nuclei with DAPI and by their ability to exclude fluorescently tagged (Fluorescein isothiocyanate) 70 kDa dextran. The cytosol was separately prepared using BHK-21 cells.

A-, B-, and C-capsid concentration. To ensure that A-, B-, and C-capsids were added to nuclei at the same concentration, the fluorescent signal intensity for each capsid type was measured with a DS-11 Spectrophotometer/Fluorometer (DeNovix, Delaware, DE, USA). The GFP-labelled capsids were excited with the laser at 470 nm and the emission spectra at 565–650 nm (for GFP emission) was recorded. The B- and C-capsids were then diluted to match the fluorescent signal of A-capsids due to the lower fluorescent signal of the latter. Finally, the 3 capsid types at equivalent concentrations were added to the isolated nuclei.

Reconstituted capsid-nuclei system. An in vitro viral HSV-1 DNA translocation system was built, in which the HSV-1 genome was released into the nucleoplasm in a homogenate solution mimicking the cytoplasm environment, see details in previously described protocol in [41]. In a typical system, rat liver cell nuclei were incubated with C-capsids (HSV-1 or GFP-labeled HSV-1), containing: (i) cytosol, (ii) BSA, (iii) ATP-regeneration system, see details in [41]. The system was incubated 37 °C for 40 min sufficient for capsid binding to nuclei. For inhibition studies, wheat germ agglutinin (WGA) was pre-incubated with the nuclei prior to addition of C-capsids [13].

Fluorescence microscopy. For fluorescence imaging of the reconstituted capsid-nuclei system, GFP-labeled HSV-1 C-capsids were used. After incubation of capsids with the nuclei as described above, the buffer system containing purified GFP-labeled C-capsids and nuclei were loaded onto cover-slips (Mab-Tek). The nuclei were stained with DAPI for 5 min before imaging. Overlay of the confocal 488 (for GFP emitted signal) and 358 (for DAPI emitted signal) channels show the localization of viral capsids onto the nucleus. Images were captured with a Nikon A1R HD laser-scanning confocal microscope. For inhibition studies with WGA, the nuclei were pre-incubated with 0.5 mg of WGA/mL for 20 min on ice before addition of GFP-labeled HSV-1 C-capsids.

Quantification of capsids bound per nucleus. For quantification of capsids bound per nucleus, 3D stacks of nuclei with capsids were collected with Nikon A1R laser-scanning confocal microscope using oil immersion Apochromat TIRF 100x (N.A. 1.49) DIC objective. The following image processing sequence was used for analysis: (1) Separation of blue and green channels and application of a median filter to increase the signal-to-noise ratio; (2) Z-projection of the image stacks using blue channel to enhance the definition of each nucleus, to locate the region of interest, and define the periphery of the nucleus used to allocate capsids; (3) Z-projection of the 3D stacks from the green channel to highlight the location of the most brilliant non-pixels (non-overlapping from each confocal slice) used to quantify the number of capsids attached around the nucleus surface. Z-projection shows both channels and exhibits the projection of all capsids on a nucleus; (4) application of a threshold to the green channel converting it to a binary image used for capsid quantification. Some nuclei had visible capsid aggregates which were converted to a number of individual capsids, using number of pixels corresponding to a single capsid at the nuclear membrane surface. Number of pixels for an individual capsid was separately determined using green channel capsid brightness profile relative to the background from at least 50 capsids. This brightness profile shows a Gaussian distribution, where width is associated with the spot diameter used to calculate capsid area based on circular object as a first approximation. We considered as bound single capsid all the spots that are into the projected nucleus surface with a thickness of one (fluorescent) capsid diameter. Thus, quantification of the total area occupied by the capsids per nucleus was followed by normalization of this value by the area occupied by one capsid.

Semi-quantitative Western blot. The relative pUL25 copy numbers were quantified by western blot. Samples were diluted in loading dye and StableDTT (final concentration 1X), then boiled for >10 min at 95 °C to denature capsid proteins. Samples were loaded onto a precast 4–12% Criterion XT Bis-Tris gradient gel, which was run at ~135 V until the dye front reached the bottom. The proteins were transferred onto a nitrocellulose membrane at 100 mA for 1 h 45 min, then blocked in Rockland NIR Blocking Buffer for at least 2 h. The membrane was incubated, rocking, at 4 °C overnight in the first 1° Antibody (Mouse α UL25, compliments of Dr. Jay Brown [A11E4], diluted 1:5000 in Rockland NIR BB + 0.1% Tween), then washed 4 times briefly and 4 times for 5 min/wash in TBST before a 1 h incubation in the dark at room temperature with the first 2° Antibody (Goat α Mouse IRDye 800 CW diluted 1:15,000 in Rockland NIR BB + 0.1% Tween-20). Finally, the blot was washed and scanned in a Li-Cor Odyssey imager with several intensities. The blot was then stripped in Restore PLUS Western Blot Stripping Buffer (Thermo Scientific #46430, Waltham, MA, USA) for 1 h at 37 °C. It was washed, reblocked in Rockland NIR Blocking Buffer for 1 h, then reprobed by an overnight incubation with the second 1° Antibody (Rabbit α UL38 [NC2, created by Dr. Fred Homa], diluted 1:5000 in Rockland NIR BB + 0.1% Tween-20). The membrane was washed, then incubated in the dark at room temperature for 1 h in the second 2° Antibody (Goat α Rabbit IRDye 800 CW, diluted 1:15,000 in Rockland NIR BB + 0.1% Tween-20). The blot was washed and scanned in the Li-Cor Odyssey imager at several intensities. The integrated pUL25 and pUL38 band intensities from the best scans were quantified with the Li-Cor Odyssey software, and the ratio of the pUL25 band intensity to the pUL38 band intensity was quantified for each sample to correct for small differences in the sample loads in each lane. Protocol provided by Jamie Belinda Huffman, University of Pittsburgh.

Electron microscopy (EM). After binding of capsids to nuclei, the samples were washed with CBB buffer. The supernatant was then removed and replaced with fixative (2.5% EM-grade glutaraldehyde and 2.0% EM-grade formaldehyde in 0.1 M sodium cacodylate buffer, pH 7.4) for 3 h at 4 °C. The fixative was then removed and replaced with 1% osmium tetroxide in buffer for 90 min. Each sample was then subjected to a 10 min buffer rinse, after which it was placed in 1% aqueous uranyl acetate and left overnight. The next day, each sample was dehydrated by using a graded ethanol series and propylenoxid. The nuclear pellets were embedded in Epon prior to cutting. Ultrathin Epon sections on grids were stained with 1% aqueous uranyl acetate and lead citrate [53]. After the grids dried, areas of interest were imaged at 120 kV, spot 3, using a Tietz 2kx2k camera mounted on a Philips/FEI (now Thermo Fisher FEI) CM200 transmission electron microscope.

## Data Availability

Data is contained within the article.
